# Documenting elimination of co-circulating COVID-19 clusters using genomics in New South Wales, Australia

**DOI:** 10.1186/s13104-021-05827-x

**Published:** 2021-11-17

**Authors:** Alicia Arnott, Jenny Draper, Rebecca J. Rockett, Connie Lam, Rosemarie Sadsad, Mailie Gall, Elena Martinez, Roy Byun, Jennie Musto, Ben Marais, Sharon C.-A. Chen, Jen Kok, Dominic E. Dwyer, Vitali Sintchenko

**Affiliations:** 1 Centre for Infectious Diseases and Microbiology Laboratory Services, NSW Health Pathology - Institute of Clinical Pathology and Medical Research, Westmead, NSW Australia; 2 Sydney Institute for Infectious Diseases, The University of Sydney, Sydney, NSW Australia; 3 Centre for Infectious Diseases and Microbiology – Public Health, Westmead Hospital, Sydney, NSW Australia; 4 Sydney Informatics Hub, Core Research Facilities, The University of Sydney, Sydney, NSW Australia; 5 Health Protection New South Wales, NSW Ministry of Health, Sydney, NSW Australia; 6 Children’s Hospital at Westmead, Sydney, NSW Australia

**Keywords:** SARS-CoV-2, Genomic epidemiology, Bioinformatics, Whole genome sequencing, Australia

## Abstract

**Objective:**

To adapt ‘fishplots’ to describe real-time evolution of SARS-CoV-2 genomic clusters.

**Results:**

This novel analysis adapted the fishplot to depict the size and duration of circulating genomic clusters over time in New South Wales, Australia. It illuminated the effectiveness of interventions on the emergence, spread and eventual elimination of clusters and distilled genomic data into clear information to inform public health action.

**Supplementary Information:**

The online version contains supplementary material available at 10.1186/s13104-021-05827-x.

## Introduction

Since the arrival of the first COVID-19 case in Australia, the Pathogen Genomics Team at New South Wales (NSW) Health Pathology’s Institute of Clinical Pathology and Medical Research (ICPMR) and the University of Sydney have implemented prospective and responsive whole genome sequencing (WGS) of confirmed SARS-CoV-2 infections. A strong positive correlation between genomics-informed clusters and epidemiologically linked cases was rapidly established, and prospective SARS-CoV-2 WGS transitioned from a novel tool with unproven relevance to an essential element of NSW’s COVID-19 public health response [1, 2]. Key to this transformation and the uptake of genomics by public health professionals has been the development and testing of novel methods to enable better visualisation and clear communication of genomic results.

**Fig. 1:**
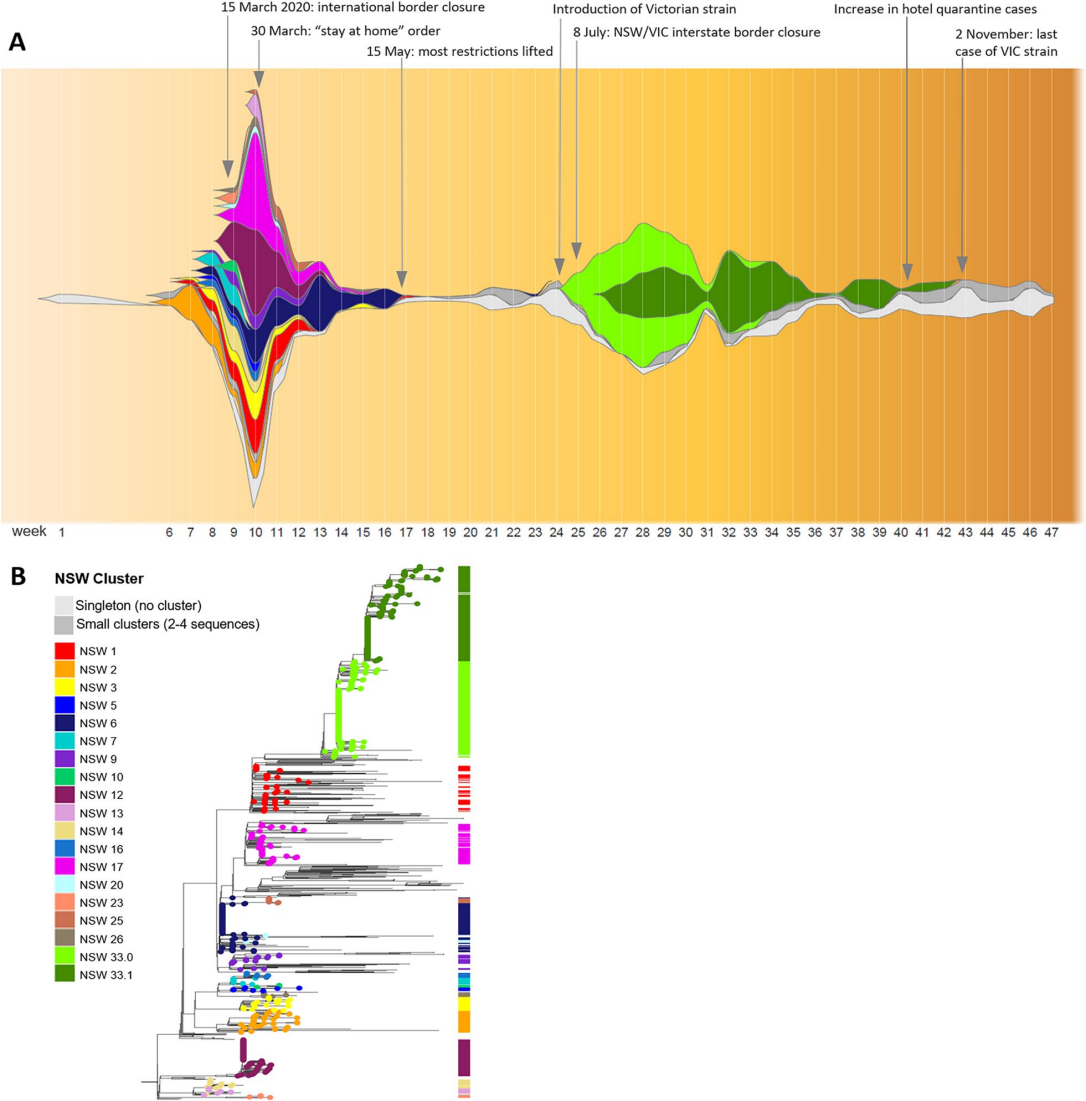
Epi-fishplot illustrating the rise and fall of SARS-CoV-2 genomic clusters in NSW over the course of the pandemic in 2020 (plotted by epidemiological week, from 22/1/2020). **a** The height of the plot at each week (vertical white line) is proportional to the total number of genomes obtained from COVID-19 cases sampled that week. The colour represents the assigned genomic cluster. The full height of the Y-axis is proportional to the largest number of sequences obtained for any time point (185 sequences, sampled during the 10^th^ week of 22–28 March 2020). This plot illustrates large number of distinct genomic clusters introduced from overseas to Australia during the initial months of the pandemic, and their rapid elimination as a consequence of border closure and lockdown measures. It also illustrates the spread and steady elimination of a “second-wave” outbreak introduced from the state of Victoria prior to interstate border closure, consisting of two related clusters, NSW33.0 and NSW33.1. **b** Classic phylogenetic tree representation of the same dataset, representing the relatedness of all sequenced cases in NSW. Clusters with ≥ 5 sequences are indicated in the metabar and coloured according to the colour scheme of the epi-fishplot (**a**). The phylogenetic tree was constructed as described previously^1^

In this correspondence we describe the adaptation and application of ‘fishplots’ to display COVID-19 genomic cluster architecture and evolution over time. The fishplot is a variant of a streamgraph, originally developed to enable visualisation of clonal tumour evolution [3]. We have adapted fishplot analysis to describe SARS-CoV-2 virus population dynamics over time, generating an “epi-fishplot” analogous to a stacked epidemiological curve (Fig. 1) [4]. This analysis simultaneously depicts the relative size and duration of circulating SARS-CoV-2 genomic clusters, defined by comparison of SARS-CoV-2 consensus sequences, over the course of the local epidemic. While a conventional phylogenetic tree represents genomic relatedness between individual cases, epi-fishplot analysis enables the timing and impact of key public health interventions to be easily identified and monitored, such as the successful elimination of circulating genomic clusters within the NSW population as a result of effective public health policies (Fig. 1).

## Main text

The state of NSW is Australia’s most populous with 8 million residents accounting for 31% of the Australian population, 65% of whom live in Greater Sydney. Since the Australian border was closed to all non-Australian travellers on March 15^th^ and Australians returning from overseas have been required to complete a mandatory 14-day quarantine in designated hotels since March 28^th^, NSW has accommodated over half of the travellers returning to Australia by air. Despite this and at the time of writing (March 28^th^, 2021), NSW accounted for just 17% (n = 5094) of Australia’s confirmed COVID-19 cases with no extensive local transmission of any strains linked to foreign travellers returning after March 15^th^. In total, overseas acquired cases restricted to hotel quarantine outweighed locally acquired cases, accounting for 59% and 41%, respectively. The multidisciplinary public health response to COVID-19 implemented in NSW is co-ordinated by the Public Health Emergency Operations Centre within the NSW Ministry of Health (NSW Health) [5]. Key to terminating local SARS-CoV-2 transmission chains has been the active case finding and contract tracing conducted by NSW Health for each laboratory-confirmed case, which included generating a SARS-CoV-2 genomic sequence to monitor its spread.

The integration of SARS-CoV-2 genomics into routine public health response has addressed key limitations of conventional epidemiological methods including poor or incorrect case recall, and confirmation of contentious or tenuous links. Furthermore, genomics has been instrumental in the timely identification of links between cases for which epidemiological links were not immediately apparent, supplementing conventional contact tracing methods and informing targeted public health resource allocation [1]. In NSW, the integration of genomics into routine public health practice includes prioritisation of clinical samples for rapid sequencing, weekly verbal and written reports to NSW Health and customised on-demand reports for urgent, high-priority cases. As of the time of writing (28th March, 2021), the Pathogen Genomics Team had generated and shared 1144 complete SARS-CoV-2 genomes representing 28% of all confirmed COVID-19 cases in NSW (Supplementary data: Additional file 1). On the basis of epidemiological information provided by NSW Health and specific single nucleotide polymorphism (SNP) profiles [1], these genomes have been classified into 53 genomic clusters. The median duration, i.e. circulation in the community, of identified genomic clusters was two weeks, although this was highly variable (range: 1–16 weeks) with clusters consisting of a median of three cases (range: 2–204 cases; Fig. 1). Epidemiological data also enabled the Pathogen Genomics Team to report which cases belong to specific transmission chains, the detail of which is described in regular reports that overlay conventional phylogenetic trees with infographics representing the supplied epidemiological data. An epi-fishplot has been generated as part of the report to provide a population-level overview of SARS-CoV-2 clusters co-circulating in the local community, illuminating the effectiveness of public health measures on the emergence, spread and eventual elimination of transmission chains within the local population (Fig. 1) [5].

Our data illustrate the two epidemiologically distinct waves of SARS CoV-2 infections experienced in NSW in 2020, the peaks of which occurred in late March and July, respectively. The first wave resulted from multiple independent introductions of genomically distinct viruses by overseas travellers prior to the closure of international borders on March 15^th^. Sustained local transmission of introduced strains was the exception and the median duration in weeks for the 17 first wave clusters identified (≥ 5 cases) was 4 (range: 1–10: Fig. 1). By mid-May 2020, local transmission of all clusters identified in the first wave had been eliminated, despite continued importation of overseas acquired cases into the NSW hotel quarantine system. This state of elimination, without significant or sustained local transmission (no clusters consisting of ≥ 5 cases) was maintained for the following two months (Fig. 1).

Genome sequencing confirmed that the second wave was seeded by a domestic importation from neighbouring state, Victoria, in early July 2020. The interstate resident travelled to NSW immediately prior to the border between the two states closing for the first time in 100 years in order to prevent spill-over from Victoria into NSW. Confirming that importation was the source of the initial second wave cluster (NSW33.0: Fig. 1), and not undetected community transmission, provided important reassurance that the NSW public health measures in place were effective. The initial infection event occurred at a large licensed venue situated in close proximity to a highway traversing the east coast of Australia, which facilitated infection of multiple individuals and enabled this strain to initiate several transmission chains amongst the local population (Fig. 1). Genome sequencing confirmed that public health measures had eliminated the NSW33.0 cluster by mid-September 2020, and the NSW33.1 subcluster by early November 2020.

Globally, the COVID-19 pandemic has necessitated extraordinary and often innovative public health responses to prevent and control widespread virus transmission. The high frequency of asymptomatic infections or subclinical disease and comparatively limited genomic diversity of circulating strains has exposed limitations of conventional epidemiological and genomic approaches when deployed in isolation to contain the spread of SARS-CoV-2.

Genomic sequencing has been recognised as a powerful public health tool that provides a unique level of resolution and oversight and addresses critical limitations of conventional epidemiological methods [6]. The epi-fish approach described above can be equally applied to other pathogens of public health importance subjected to integrated prospective genomic surveillance. The successful integration of genomics into routine public health response relies on a strong working partnership between public health practitioners and laboratory professionals, with evidence synthesis and visualisation in order to distil complex genomic data into the information which can guide and benchmark public health actions.

## Limitations

A consensus genome sequence could not be obtained from every confirmed case of COVID-19 detected in NSW during the study period. Sequences were reliably recovered from samples with a diagnostic PCR Ct value ≤ 30. However, the proportion of sequenced cases (28%) was high compared to that reported by most countries around the world [7] and more than sufficient to achieve the primary goal of this study which was to demonstrate the application and utility of the Epifish package.

## Supplementary Information

**Additional file 1.** SARS-CoV-2 genome sequences included in the visualisation of COVID-19 activity in New South Wales, Australia.

## Data Availability

The consensus genome sequences included in this study are all available from GISAID (www.gisaid.org). A full list of genomes and their corresponding IDs can be found in the Supplementary data (Additional file 1). The epifish package for R, including a tutorial, is available from Github (https://github.com/learithe/epifish). The figure was produced using RStudio v1.2.5001–1.3.1056 and R v3.6.1, with the epifish package v1.0 [4], the fishplot package v0.5, [3], and the epifish dependencies “lubridate” (v1.7.4–1.7.9), “dplyr” (v0.8.3–1.0.2) and “tidyr” (v1.0.0) from the tidyverse [8].

## Abbreviations

NSW: New South Wales
WGS: Whole genome sequencing
ICPMR: Institute of Clinical Pathology and Medical Research
SNP: Single nucleotide polymorphism
PCR: Polymerase chain reaction
Ct: PCR cycle threshold
